# Report of Deaths in the City of Buffalo, for the Month of October, 1864

**Published:** 1864-12

**Authors:** Sandford Eastman

**Affiliations:** Health Physician


*Report of Deaths in the City of Buffalo, for the month of October*, 1864.

Males 63, females 58. Locality—City at large 104, Hospital of Sisters of Charity 5, Buffalo General Hospital 2, Catholic Foundling Asylum 4, Catholic Sisters of Good Shepherd 1, Erie County Alms House 5. Total 121.

By whom certified.—By Regular Physicians at Public Institutions 16, by regular physicians in city at large 58, by irregular practitioners 17, by coroner 9, by undertakers 21.

Causes of Death.—Accident 4, do. drowning 3, angina pectoris 1, brain, congestion of 1, bronchitis 1, consumption 14, convulsions 5, croup 10, croup diphtheritic 3, debility 3, dentition 2, diabetes mellitus 1, diarrhœa 7, disease of heart 3, disease of womb 1, disease of stomach 1, diphtheria 6, dropsy general [illegible], dysentery 5, epilepsy 1, erysipelas 1, fever conjestive 2, do. remittent 1, do. scar et 2, do. typhoid 8, inflammation of bowels 5, do. brain and meninges 3, do. lungs 1, do. lungs typhoid 3, do. pericardium 1, jaundice 1, marasmus 3, old age 3, paralysis 1, premature birth 2, pyæmia 1, rheumatism 2, unknown 3. Deaths from diseases m. 60, f. 57, total 117. In addition to the above, 4 still-born were reported in the city.

SANDFORD EASTMAN, M. D., Health Physician.

